# The role of initial ^18^F-FDG PET/CT in the management of patients with suspected extramedullary plasmocytoma

**DOI:** 10.1186/s40644-018-0152-x

**Published:** 2018-05-15

**Authors:** Linqi Zhang, Xu Zhang, Qiao He, Rusen Zhang, Wei Fan

**Affiliations:** 1 Department of Nuclear Medicine, Affiliated Cancer Hospital&Institute of Guangzhou Medical University, Guangzhou, 510095 People’s Republic of China; 2 Department of Nuclear Medicine, Sun Yat-sen University Cancer Center, State Key Laboratory of Oncology in South China, Collaborative Innovation Center of Cancer Medicine, Guangzhou, 510060 People’s Republic of China; 3 Department of Nuclear Medicine, The First Affiliated Hospital of Sun Yat-sen University, Guangzhou, 510080 People’s Republic of China

**Keywords:** Extramedullary plasmacytoma, ^18^F-FDG, PET/CT, Management

## Abstract

**Background:**

Extramedullary plasmacytoma (EMP) is a plasma cell malignancy that originates in soft tissues without evidence of systemic spread, and its management differs from other plasma cell neoplasms. The purpose of the present study was to evaluate the role of initial ^18^F-FDG PET/CT in the management of patients with clinical suspected EMP.

**Methods:**

^18^F-FDG PET/CT scans performed in 21 patients (M/F = 12/9, mean age 51.1 ± 15.3 years) with clear suspicion of EMP from 2006 to 2015 were analysed retrospectively. The detection of new lesions and the change in treatment were evaluated.

**Results:**

PET/CT detected new lesions in 38.1% (8/21) of patients with 17 lesions, and lymph nodes were the most common site, accounting for 70.6% (12/17) of all lesions, followed by bone (*n* = 2), and less frequently, breast (*n* = 1), lung (*n* = 1), and stomach (*n* = 1). These findings resulted in treatment changes in 7 patients with EMP. Among these, 4 patients had major treatment changes and 3 patients had minor changes. Of the 21 patients, progression to MM was observed in 8 patients (8/21, 38.1%). In univariate analysis, tumour size > 4 cm and partial response (PR) after treatment were significant prognostic factors for Progression-free survival (PFS).

**Conclusions:**

Our data indicated that ^18^F-FDG PET/CT is helpful in the detection of additional lesions throughout the body, including lymph node involvement and distant additional lesion, which may have resulted in treatment change.

## Background

Plasma cell tumours are classified into two main groups: multiple myeloma (MM) and solitary plasmacytoma (SP). Solitary plasmacytoma (SP) is a rare plasma cell malignancy without evidence of systemic spread. It accounts for 5–10% of all plasma cell neoplasms and comprises two subsets, including solitary extramedullary plasmacytoma (EMP) and solitary plasmacytoma of bone (SBP). EMP accounts for 3 to 5% of all plasma cell neoplasms, a percentage that is less than that reported for SBP [1–3]. EMP may originate in soft tissues throughout the body, although it most frequently occurs in the upper respiratory tract and oral cavity. It is three times more common in males than in females, and the most frequent age of patients is after 50 years [4]. However, EMP evolves into MM less frequently than SPB. As a result, EMP demonstrates better prognosis compared with SBP. Tumor size, lymph node metastasis and distant lesion involvement have been identified as clinical factors affecting EMP progression to MM [5–7]. Considering the poor prognosis of MM, the accurate staging of EMP and exclusion of the possibility of systemic involvement are important steps in management of these patients.

However, there is no current consensus on optimal imaging modalities for evaluating EMP. A skeletal X-ray survey and whole-body bone scan (WBS) were the first investigations recommended for the evaluation of MM, although skeletal X-ray has poor sensitivity regarding specific localisations, such as those in the vertebrae and pelvic bones, and WBS has low sensitivity in osteolytic lesions. Whole-body computed tomography (CT) can provide additional information but is not systematically performed and has low sensitivity for detecting nodal disease smaller than 1 cm. Whole-body MRI scans have also been proposed as an alternative for assessing EMP, but this method does not accurately assess the activity of disease [8–10]. ^18^F-fluorodeoxyglucose positron emission tomography/computed tomography (^18^F-FDG PET/CT), which combines an anatomical evaluation with an assessment of the metabolic activity of lesions, should be particularly useful in this context. Although the role of PET/CT with proven performance in the evaluation of MM has been well documented in the literature, the literature regarding the use of ^18^F-FDG PET/CT in EMP has only been reported in individuals or a small number of cases, and the precise role of PET/CT in EMP has not yet been established [11–17]. The aim of this study was to evaluate the role of ^18^F-FDG PET/CT in the staging and management of EMP in a much larger cohort of patients.

## Methods

### Patients

All patients referred to 18F-FDG PET/CT with pathologically proven plasmacytoma during a 10-year period (2006–2015) were registered. Data were retrieved retrospectively from our department’s searchable database and consisted of all scans performed with an assigned indication of “Solitary plasmacytoma or MM”. Each referral was reviewed manually regarding the selection of patients with suspected EMP. The following criteria were used for inclusion: (1) biopsy of tissue confirmed lesions with neoplastic-appearing plasma cells; (2) conventional imaging had no evidence of diffused bone involvement; (3) bone marrow biopsy ensured plasma cell infiltration not exceeding 5% of all nucleated cells; (4) absence of hypercalcaemia, significant cytopenia, renal dysfunction, prior treatment for plasma cell neoplasm, or second malignancy; and (5) low serum monoclonal protein (M-protein) concentration. Patients were excluded if they had a history of malignancy or had accepted any treatment. This study was approved by our institution’s Institutional Review Board, and due to the retrospective nature of the study, the requirement for written informed consent was waived.

### Data collection

The electronic medical records of all patients with clinical suspected EMP were reviewed to obtain demographic data and indication for performing the ^18^F-FDG PET/CT. Changes were compared in the management of EMP described in the patient’s medical records before and after the ^18^F-FDG PET/CT exam. The ^18^F-FDG PET/CT scan results were compared with the histological data and clinical follow-up as well as with the conventional imaging available for each patient.

### PET/CT imaging

^18^F-FDG PET/CT was performed using PET/CT scanners (Discovery ST 16, General Electric Medical System, Milwaukee, Wisconsin, USA). After patients had fasted for at least 6 h, they were injected with FDG (dose: 0.15 mCi/kg) and the images were acquired after an approximate uptake phase time of 68 ± 12 min. PET/CT images were typically obtained from the base of the brain through the proximal thigh and were reconstructed into three planes. An un-enhanced CT scan was obtained for attenuation correction and anatomical correlation.

### Image analysis

Two experienced nuclear medicine physicians with more than 5 years of experience in PET/CT imaging retrospectively evaluated the ^18^F-FDG PET/CT studies. The conventional images (CT, MRI and WBS) were independently interpreted by two experienced nuclear medicine physicians together with a diagnostic radiologist. All physicians were unaware of the other imaging information at the time of review. In cases of discrepancy, a consensus was obtained by a joint reading. A PET/CT study was considered positive if there was a focal abnormality with FDG uptake greater than background, which was not explained by physiological distribution or another benign process. ^18^F-FDG PET/CT performance was appraised by considering the histological data as the gold standard. When the histological data were not available, a combination of imaging results and clinical follow-up was considered. A PET/CT study was considered negative when ^18^F-FDG uptake was only observed in the primary tumour and/or lymph node findings by conventional images and there were no other lesions to suggest malignancy identified on the remainder of the body.

Changes in therapy were classified as “major” (change in adding systemic therapy) or “minor” (change in enlarging lymphatic field irradiation). Furthermore, follow-up in all patients starting at the time of the first staging until the last observation or death was documented.

### Statistical analysis

Categorical data are expressed as numbers and frequency (%). Continuous data are expressed as the mean and standard deviation. Progression-free survival (PFS) was obtained from the date of treatment initiation to the date when local relapse and/or progression to MM occurred. PFS were analyzed with Kaplan-Meier curves and log-rank test was applied for statistical comparison of independent subgroups. All statistical tests were performed using SPSS Statistics 17.0 (SPSS Inc., Chicago, IL, USA) software.

## Results

### Patient population

In total, 769 patients were diagnosed with pathologically proven solitary plasmacytoma or MM and underwent ^18^F-FDG PET/CT from 2006 to 2015 at our center. Of these patients, 21 patients (one patient presented with resection of known primary lesion) with clear clinical suspected EMP diagnosed by laboratory tests and conventional imaging (CT, MRI and WBS) before undergoing ^18^F-FDG PET/CT were included in this study.

Relevant characteristics of all 21 patients are presented in Table 1. The mean age of the patients at diagnosis with plasmacytoma was 51.1 ± 15.3 years (ranged from 27 to 92 years), with the majority of the patients (52.4%, 11/21) over the age of 50 years. The male to female ratio was 12:9. The level of serum M-protein was elevated in 5 out of 21 (23.8%) patients. The median interval between PET/CT with bone marrow biopsy was 9 days (range 1 – 17 days), and 2 patients (9.5%) showed plasma cell infiltration, but not exceeding 5%. Two patients (9.5%) showed elevated beta-2 microglobulin (β^2^-MG) levels, but all patients were found to demonstrate less than 3.5 mg/dL. The median interval between PET/CT with conventional imaging (CT, MRI and WBS) was 5 days (range 0 – 27 days). Before ^18^F-FDG PET/CT, 4 patients exhibited primary lymph node EMP, 14 patients were considered to have only one site of primary plasmacytoma as assessed by standard imaging, and 2 patients presented regional lymph node involvement. The average tumour size, measured as the greatest lesion dimension of the 21 patients, was 4.2 cm (range 1.4–9.2): 9 patients had lesion > 4 cm, and 12 patients had lesion ≤4 cm. The median SUV_max_ uptake of the primitive lesion was 5.75 (range 2.3–21.9): 11 patients had SUV_max_ > 5.5, and 9 had SUV_max_ ≤ 5.5. For 1 patient (case 21), the PET/CT was performed after surgical excision of the primary lesion in the right salivary gland.

**Table 1:** Laboratory and imaging data in all patients with suspected EMP

Case	Type of M-protein	Serum M-protein(g/L)	Serum β^2^-MG(g/l)	Plasma cells in BM	Extent evaluated by conventional imaging	Post-PET/CT changes
1	NEG	NEG	1.76	NEG	Single primary EMP in left nasal cavity	No
2	NEG	NEG	1.90	NEG	Single primary EMP in nasopharynx with left cervical LN metastasis	Detected more lymph node metastasis(bilateral cervical LN metastasis, *n* = 2)
3	NEG	NEG	1.50	NEG	Single EMP in nasopharynx	No
4	NEG	NEG	1.88	NEG	Single EMP in left nasal cavity	No
5	IgA	16.4↑	2.25	2.5%	Primary EMP of LN in left supraclavicular area	Detected more lymph node involvement in mediastinal area(*n* = 1)
6	NEG	NEG	1.82	NEG	Single primary EMP in right lung	No
7	NEG	NEG	1.78	NEG	Single primary EMP in left nasal cavity	No
8	NEG	NEG	1.64	NEG	Single primary EMP in left nasal cavity	Bone involvement (left pubis, *n* = 1) was identified
9	IgG	22.00↑	2.55↑	NEG	Single primary EMP in left maxillary sinus	Bone involvement (right iliac bone, *n* = 1) was identified
10	NEG	NEG	1.72	NEG	Single EMP in nasopharynx	No
11	NEG	NEG	1.98	NEG	Single primary EMP in right nasal cavity	No
12	IgG	16.3↑	1.43	0.4%	Single primary EMP in right thyroid	A second primary EMP (left breast, *n* = 1)
13	NEG	NEG	1.66	NEG	Single primary EMP of small intestine with locoregional LN metastasis	Detected more lymph node metastasis (*n* = 2)
14	NEG	NEG	1.66	NEG	Single primary EMP in left oropharynx	No
15	NEG	NEG	1.70	NEG	primary EMP of LN in retroperitoneal lymph nodes	No
16	NEG	NEG	1.78	NEG	primary EMP of LN in left hilar	No
17	NEG	NEG	1.82	NEG	Single primary EMP in right lung	No
18	NEG	NEG	2.04	NEG	Single primary EMP in right maxillary sinus	Multiple EMP (right lung, right hilar, gastric body, *n* = 3) and lymph node involvement (*n* = 1) were identified
19	IgG	26.1↑	1.74	NEG	Single primary EMP in right lung	No
20	IgG	23.00↑	3.26↑	NEG	Single primary EMP in left inguinal, bilateral iliac area	Detected more lymph node involvement in retroperitoneal area (*n* = 6)
21	NEG	NEG	2.27	NEG	Surgical excision of single primary EMP in right salivary gland	No

*M* male, *F* female, *M-protein* monoclonal protein, *NEG* negative, *EMP* extramedullary plasmocytoma, *β*^*2*^*-MG* beta-2 microglobulin (normal range, 1.16-2.52), *BM* bone marrow (normal range, <5%)

### Change in stage after ^18^F-FDG PET/CT

**Fig. 1:**
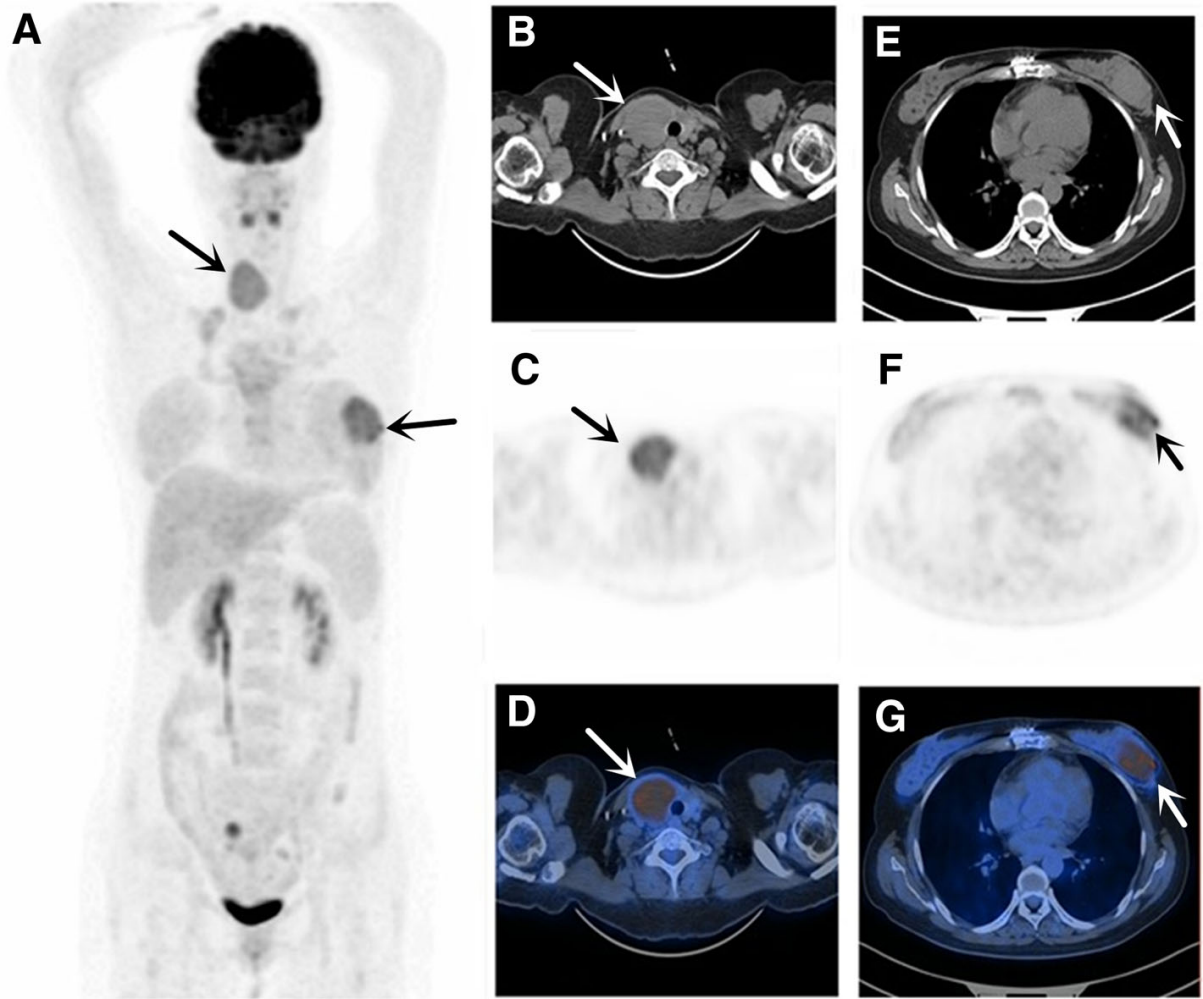
Case 12, **a** 38-year-old woman found to have a mass in the right thyroid, and ultrasound-guided fine-needle aspirations (USG-FNA) revealed EMP of the thyroid. Without evidence of systemic spread according to other clinical examination, suspicion of EMP was made. ^18^F-FDG PET/CT was performed for further evaluation. Maximum intensity projection (**a**), CT, PET and fused PET/CT images (**b-g**) showed extensive hypermetabolic lesions involving the right thyroid, and an additional lesion in the left breast (surgery confirmed EMP) was also identified. Due to detection of new lesions in the breast, more invasive therapy was given, including enlarged surgical region, enlarged field irradiation and systemic therapy, instead of resection of thyroid plasmacytoma combined with RT alone

**Fig. 2:**
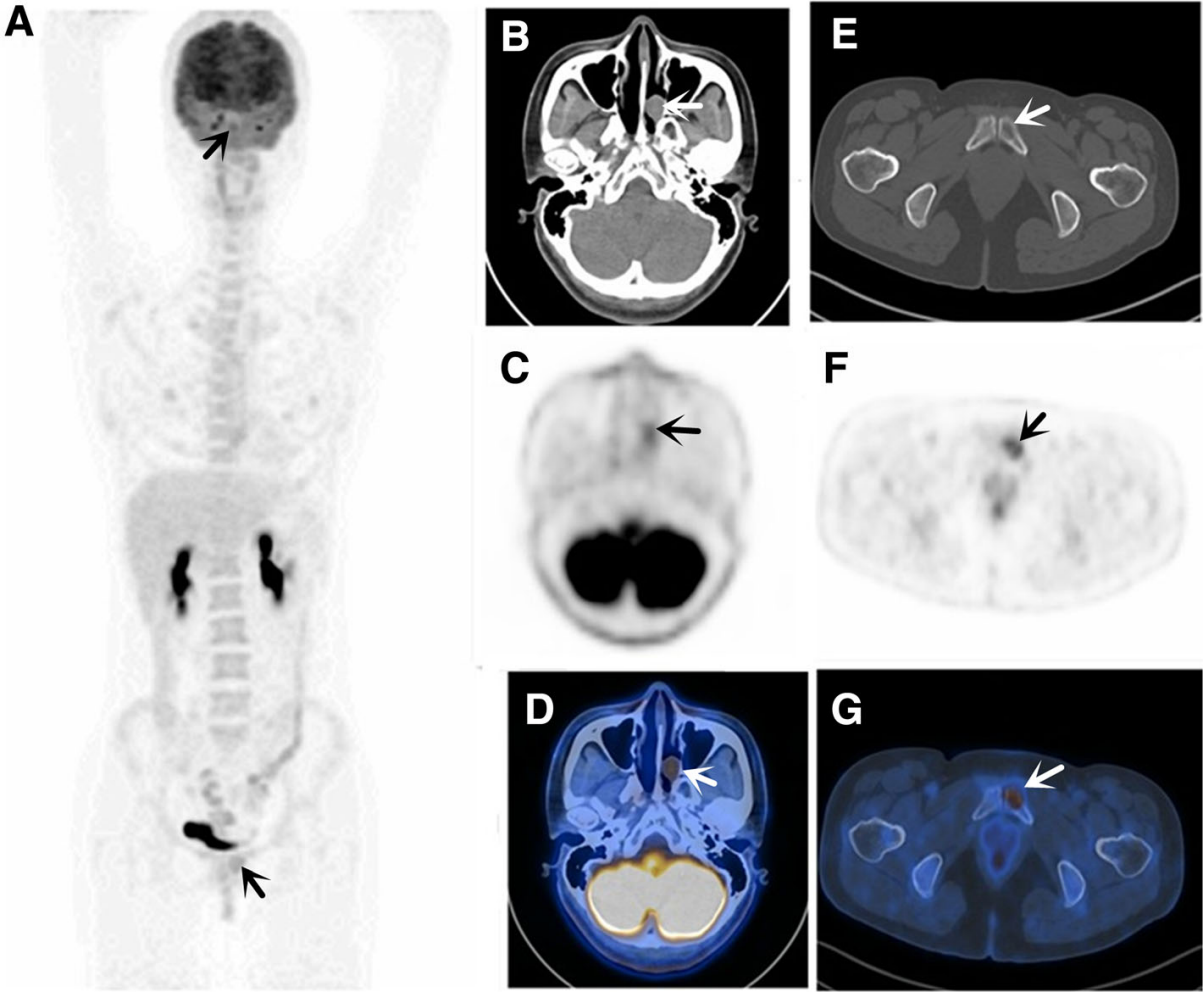
Case 8, a 36-year-old woman presented with nasal obstruction for three months, in whom the nasal endoscope showed the plasmacytoma in the left nasal cavity. Without evidence of systemic spread according to other clinical examination, suspicion of primary EMP of nasal cavity was made. ^18^F-FDG PET/CT was performed for further evaluation. CT (**b**), PET (**c**) and fused PET/CT (**d**) images showed ^18^F-FDG uptake in EMP of the nasal cavity. Another osteolytic lesion in the left pubis was identified by PET/CT (**a, e-g**). A biopsy of the pubis lesion was performed, which showed neoplastic-appearing plasma cells involved in the bone. Due to detection of new lesions in the bone, more invasive therapy was given, including enlarged field irradiation and systemic therapy, instead of resection of nasal plasmacytoma combined with RT alone

Prior to conducting the ^18^F-FDG PET/CT, all patients had at least one regionally enhanced CT or MRI for diagnosis and staging primary EMP. Pelvis MRI was performed in 7 patients and WBS in 16 patients, to determine whether there was any bone involvement. Changes in stage between conventional imaging and PET/CT are shown in Table 1. PET/CT and conventional imaging were consistent with regard to staging in 61.9% (13/21) but differed in 38.1% of cases, as ^18^F-FDG PET/CT detected new lesions in 8 patients. In 2 patients, ^18^F-FDG PET/CT detected additional primary EMP in regions where conventional imaging (CT or MRI) was not performed. One patient (case 18) presented a primary lesion in the right maxillary sinus (confirmation by biopsy), which was identified by enhanced CT and PET/CT, demonstrating intense ^18^F-FDG uptake in the right maxillary sinus, as well as the gastric body (*n* = 1, confirmation by gastroscope), lung (*n* = 1), and right hilar lymph nodes (*n* = 1). In another patient (case 12, Fig. 1) in whom ultrasound-guided fine-needle aspirations (USG-FNA) revealed EMP of the thyroid, a second primary EMP in the left breast (*n* = 1, confirmation by surgery) was identified by PET/CT. For 2 patients, PET/CT found two sites of SBP (both confirmed by biopsy), which were missed on WBS. One (case 8, Fig. 2) SBP was located in the left pubis, and another (case 9) SBP was found in the iliac bone. In 4 patients with suspected primary EMP of lymph nodes, ^18^F-FDG PET/CT detected more lymph node involvement in 2 patients. For 1 patient presenting with enlarged left supraclavicular lymph nodes (biopsy-confirmed EMP), PET/CT demonstrated another enlarged lymph node in the mediastinal area (*n* = 1) with increased ^18^F-FDG uptake in regions where conventional imaging (CT or MRI) was not performed. Another patient (case 14) presented primary EMP of the lymph nodes involving the inguinal and iliac, and PET/CT detected 6 additional lesions (lymph nodes short-axis less than 1 cm) due to increased ^18^F-FDG uptake. In 2 patients (patients 10 and 13) with primary EMP in the nasopharynx and intestine, local lymph node metastasis was present and more lymph node metastasis (*n* = 4, lymph node short-axis less than 1 cm) was identified by ^18^F-FDG PET/CT.

To summarise, PET/CT detected new lesions in 8 patients with 17 lesions, with the lymph node being the most common site, accounting for 70.6% (12/17) of lesions, followed by bone (*n* = 2), and less frequently, breast (*n* = 1), lung (*n* = 1), and stomach (*n* = 1). Three out of 12 (25%) lymph nodes and 5 other additional distant lesions had histological or cytological confirmation. The remaining 75% of lymph nodes (9/12) were confirmed with radiological follow-up after treatment. When clinical follow-up was used for validation, the median length of follow-up was more than 6 months (22–76 months).

### Change in management after ^18^F-FDG PET/CT

The final management plans for the 21 patients after ^18^F-FDG PET/CT were as follows: All patients were treated with radiotherapy alone (*n* = 12) or combined with surgery (*n* = 5) or chemotherapy (*n* = 2) or both (*n* = 2).

The effect of the PET/CT results on treatment planning was as follows (detailed in Table 2): findings resulted in treatment changes in 7 patients. Major changes: In 4/21 patients (19%), the use of PET/CT had a substantial effect on management plans due to the finding of additional distant lesions, resulting in treatment with systemic chemotherapy. The chemotherapy regimens for the 4 patients were as follows: MP regimen in 2 patients (cases 8 and 18), consisting of melphalan (8 mg/m2, days 1 − 4) and prednisone (60 mg/m^2^, days 1 − 4); and VAD in 2 other patients (cases 9 and 12), consisting of vincristine (0.4 g/d, days 1 − 4), epirubicin (9 mg/m^2^/d, days 1 − 4), and dexamethasone (40 mg/d, days 1 − 4, 9 − 12, and 17 − 20).

**Table 2:** Patients in whom PET/CT showed additional lesions and treatment changes

Case Pre-PET/CT treatment planning	Post-PET/CT treatment planning	Comment
Major treatment changes		
8 Resection of nasal plasmacytoma combined with RT	Resection of nasal plasmacytoma plus RT for nasal cavity and left pubis lesion, chemotherapy was given	More invasive therapy was given, including enlarged field irradiation and systemic therapy
9 RT for primary EMP in nasopharynx	RT for EMP of nasopharynx and iliac bone lesion, chemotherapy was given	More invasive therapy was given, including enlarged field irradiation and systemic therapy
12 Resection of thyroid plasmacytoma combined with RT	Resection of thyroid and breast plasmacytomas, RT for surgical margin combined with concomitant chemotherapy	More invasive therapy was given, including surgical region, enlarged field irradiation and systemic therapy
18 RT for primary EMP in right maxillary sinus	RT for primary EMP in right maxillary sinus combined with concomitant chemotherapy	Systemic therapy was given
Minor treatment changes		
2 RT for primary EMP in nasopharynx and left cervical lymph node metastasis	RT for primary EMP in nasopharynx and bilateral cervical lymph node metastasis	Only enlarged lymphatic field irradiation
5 RT for left supraclavicular area	RT for supraclavicular and mediastinal area	Only enlarged lymphatic field irradiation
13 Resection of intestinal plasmacytoma combined with RT	No change	
20 RT for left inguinal, bilateral iliac area	RT for left inguinal, bilateral iliac and retroperitoneal area	Only enlarged lymphatic field irradiation

*RT* radiotherapy, *EMP* extramedullary plasmocytoma

Minor changes: In 3 out of 21 patients (14.3%), the use of PET/CT had some degree of effect on clinical decisions about treatment due to the detection of more lymph node involvement, resulting in treatment with enlarged lymphatic field irradiation. In 14 out of 21 patients (66.7%), including 1 patient (case 13) with the finding of an additional lesion, the addition of the PET/CT findings led to no difference in treatment planning.

^18^F-FDG PET/CT and conventional imaging were performed to evaluate therapeutic response (after first-line treatment), which showed complete response (CR) in 13 patients and partial response (PR) in 8 patients.

### Outcomes

No patient was lost to follow-up. At the time of the data analysis, the median follow-up for all patients was 45.9 ± 24.5 months (range, 15–99 months). Progression to MM was observed in 8 patients (8/21, 38.1%). Of the 8 patients, 3 patients had local recurrences, which then evolved to MM. The 5 other patients directly evolved to MM. The mean time of progression to MM was 32.3 ± 29.6 months (range, 4–85 months) after the first-line treatment. Of the 8 patients, 2 patients died due to MM-related diseases during the follow-up period. Nineteen patients were alive at the time of the analysis, and 13 (61.9%) patients showed no evidence of disease (NED).

**Fig. 3:**
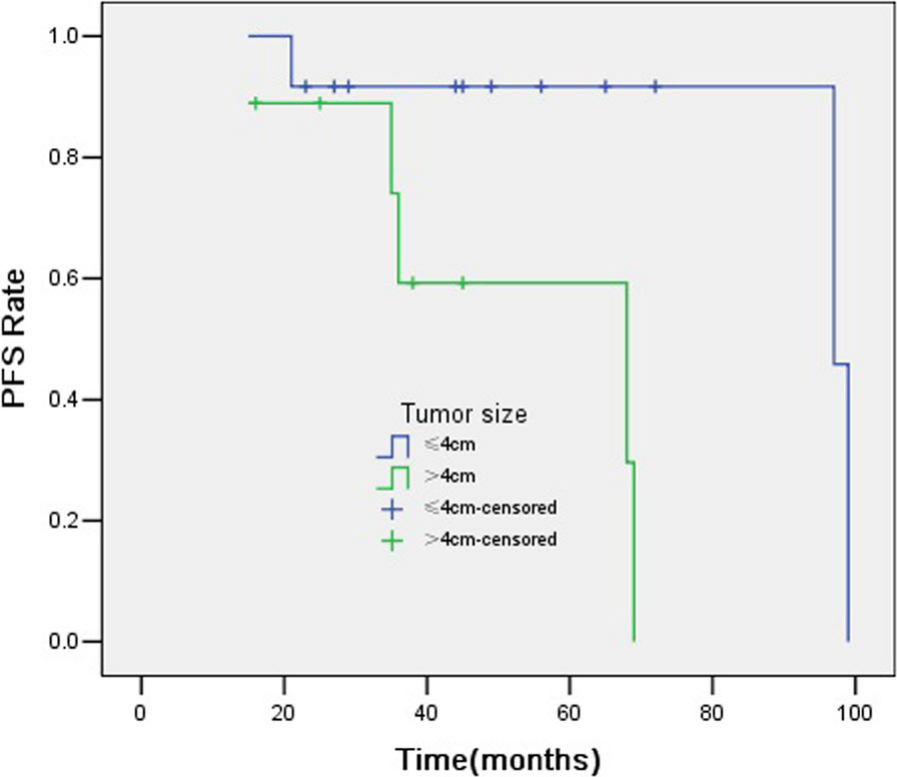
Kaplan-Meier curves for PFS showing significant difference is seen between patients classified as tumour size ≤4 cm and tumour size > 4 cm (log-rank test, *p* = 0.020)

**Fig. 4:**
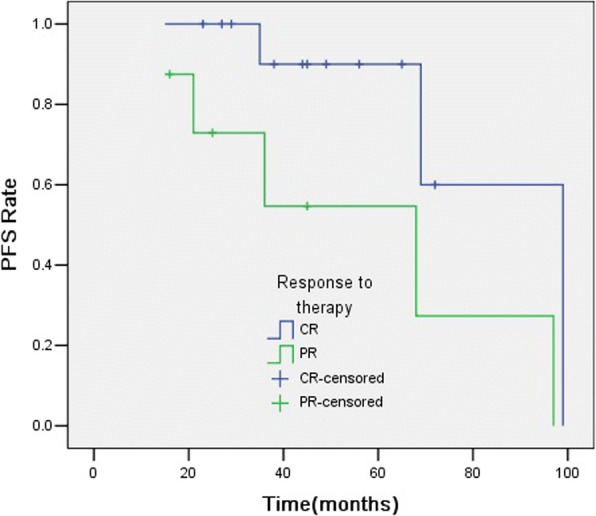
Kaplan-Meier curves for PFS showing significant difference is seen between patients classified as CR and PR after treatment (log-rank test, *p* = 0.040)

In univariate analysis (detailed in Table 3), tumour size > 4 cm (Fig. 3) and PR after treatment (Fig. 4) were significant prognostic factors for PFS (*p* = 0.020 and 0.040). The other factor, such as age, gender, tumour number, and SUV_max_ were not significant predictors for PFS.

**Table 3:** Univariate analysis of predictive factors for PFS

Factor	No. of patients	Progression rate,%	*p* value
Gender			
Male	7/13	53.8%	0.475
Female	1/8	12.5%	
Age			
≤ 50	4/11	36.3%	0.835
> 50	4/10	40.0%	
Tumor size(cm)			
≤ 4	3/12	25.0%	0.020*
> 4	5/9	55.6%	
Tumor number			
SP	5/18	27.8%	0.114
MSP	3/3	100%	
SUV_max_			
≤ 5.5	2/9	22.2%	0.627
> 5.5	6/11	54.5%	
Response to therapy			
CR	3/13	23.1%	0.040*
PR	5/8	62.5%	

*p<0.05

## Discussion

Currently, the staging system for EMP remains to be defined. According to the Durie and Salmon staging system, EMP is considered stage I myeloma. Wiltshaw classified EMP into three clinical stages: patients presenting with a single extramedullary site were considered to be in stage I; patients with regional lymph node involvement were considered to be in stage II; and patients with distant lesion (metastasis or multiple solitary plasmacytoma) were considered to be in stage III, although it is no longer a SP [18]. In our center, a previous study conducted by Zhu et al. also classified EMP into three clinical stages according to independent poor prognostic factors (lymph node metastasis and a larger primary tumour) for overall survival [7]. Although EMP exhibits the same etiopathogenesis as MM, it differs from MM in that clinical presentations and the treatment approach differ since solitary lesions are amenable to local radiation therapy (RT) [19, 20]. Despite curative treatment, progression to MM was also found in 10-20% of patients with EMP, which is a challenging problem due to the subsequent worsening of overall survival [2, 5, 21]. Therefore, accurate identification of all sites of lesion and the initiation of an appropriate treatment plan are essential.

Thus far, only a few case reports and articles based on small sample sizes (*n* = 1-9) have documented the usefulness of ^18^F-FDG PET/CT in the management of EMP [11–17]. In our study, PET/CT showed additional lesions in 8/21 (38.1%) patients with EMP. More precisely, this approach identified 17 additional lesions that not only involved the lymph nodes and bone but also the breast, lung and stomach. Our results are consistent with those of a study conducted by Salaun et al. with a cohort of 20 patients with SP (EMP = 4) in which ^18^F-FDG PET/CT detected more lesions in 10/20 patients (50%) with 18 areas (bone: 8 patients, soft tissue: 2 patients), but the effect on management was not evaluated in their study [16]. Our study was also consistent with another previous study in which ^18^F-FDG PET detected 20 additional plasmacytoma lesions in 5 patients but one false negative in 1 patient and an indeterminate in another patient due to small or slightly active lesions. Importantly, PET images were not coupled with CT scans in their study [15]. In our study, we used a hybrid system of PET integrated with CT, which allows for the precise detection of small and/or slightly active lesions. Such lesions are not easily recognised or differentiated from soft tissue lesions when using PET alone. At present, the diagnostic criterion for lymph node involvement based on contrast-enhanced CT images is the presence of the lymph node short-axis > 1 cm but with low specificity. In addition, using fused images, where each hypermetabolic lesion was concordant with the morphology of a lesion on the corresponding CT image, we could directly confirm the reliability and specificity of our ^18^F-FDG PET/CT findings in all patients.

These findings resulted in treatment changes in 7 patients, which had major changes in 4 patients and minor changes in 3 patients. Indeed, the identification of a distant additional lesion was the factor with a major impact on the management of these patients. All 3 patients accepted systemic chemotherapy to prevent the progression to MM due to the PET/CT findings. For example, case 12 was admitted with thyroid plasmacytoma as revealed by USG-FNA. PET/CT identified a second soft-tissue mass in the left breast, which led to a more invasive approach, including resection of the thyroid and breast mass combined with RT, plus systemic chemotherapy, rather than resection of thyroid plasmacytoma combined with RT alone. Finding more lymph nodes was the factor most often found to underlie the minor impact on management plans from PET/CT findings. A previous study demonstrated that PET/CT had a better diagnostic performance than conventional imaging for interpreting lymph node involvement [22]. PET/CT identified 12 additional lymph nodes in 4 patients and resulted in enlarging lymphatic field irradiation in 3 patients. Only one retrospective study of suspected SP, which included only patients with suspected EMP and 11 patients with SBP, evaluated the role of PET/CT in management plans [14]. In that study, Kim et al. evaluated the usefulness of staging of ^18^F-FDG PET/CT with suspected EMP and found that PET/CT could influence management in 6 of 17 (35.3%) patients. Overall, the use of PET/CT increased appropriate curative interventions in these patients.

In our series, the upper airway was the most common site involved in EMP, which is consistent with other studies [4, 17, 23, 24]. However, it was also simultaneously found in contiguous sites, confirming that the disease tended to spread locally. The uncommon localisations in the lung, thyroid gland and breast observed in this study proved that the disease might affect any part of the body. Another salient finding of our study was the identification by PET/CT of multiple solitary plasmacytoma in 5 out of 21 patients, which is more frequent than previously reported in SP. Major advantages of ^18^F-FDG PET/CT were the ability to perform a full body examination and the potential to detect medullary and extramedullary lesions in one single examination. In 2000, Kato et al. reported a patient with EMP and ^18^F-FDG PET showed early detection of bone marrow involvement [10]. In our study, ^18^F-FDG PET found bone marrow involvement in two patients, which was consistent with their study.

Factors which can predict EMP progression to MM are not clearly identified. Previous studies have showed that tumour size, increased ^18^F-FDG uptake and distant lesion involvement have been identified as predictive factor for SP progression to MM [5–7]. However, most of cases in their study were patients with SBP, relatively small numbers of patients with EMP. In present study, we observed that tumour size > 4 cm and PR after treatment were significant prognostic factors for PFS, SUV_max_ were not significant predictors for PFS. Studies with a larger sample size are required to validate these observations. In addition, ^18^F-FDG PET/CT combines with anatomical and functional imaging could be a potential useful modalities in assessing response to therapy in EMP, which has been reported in SBP [25].

Our study presents a number of limitations that call for caution in the interpretation of our results. One of the limitations of this study was the small number of patients included in this study. We are aware that a definite conclusion regarding the role of ^18^F-FDG PET/CT in the management of EMP can only be drawn from a study by recruiting a large number of patients. However, it is very difficult to enrol a large number of patients at a single center due to the very low incidence of EMP. The second major limitation of our study was due to its retrospective nature in which a referral bias cannot be excluded. Finally, not all lesions detected by ^18^F-FDG PET/CT were histologically confirmed. This was not ethically and technically feasible. However, all lesions without histological evidence were examined by imaging and clinical follow-up. Future multicentre prospective studies addressing these shortcomings are warranted.

## Conclusion

Our study aimed to illustrate the potential clinical value of initial ^18^F-FDG PET/CT in the management of clinical suspected EMP. Our data indicated that ^18^F-FDG PET/CT is helpful in the detection of additional lesions throughout the body, including lymph node involvement and distant additional lesion, which may result in treatment change.

## Abbreviations

^18^F-FDG PET/CT: ^18^F-fluorodeoxyglucose positron emission tomography/computed tomography
EMP: Extramedullary plasmacytoma
MM: Multiple myeloma
SBP: Solitary plasmacytoma of bone;
SP: Solitary plasmacytoma
WBS: Whole-body bone scintigraphy
